# Characterizing Uncertainty in Machine Learning for Chemistry

**DOI:** 10.1021/acs.jcim.3c00373

**Published:** 2023-06-20

**Authors:** Esther Heid, Charles J. McGill, Florence H. Vermeire, William H. Green

**Affiliations:** ‡ Department of Chemical Engineering, Massachusetts Institute of Technology, Cambridge, Massachusetts 02139, United States; ¶ Institute of Materials Chemistry, TU Wien, 1060 Vienna, Austria; § Department of Chemical and Life Science Engineering, Virginia Commonwealth University, Richmond, Virginia 23284, United States; ∥ Department of Chemical Engineering, KU Leuven, Celestijnenlaan 200F, B-3001 Leuven, Belgium

## Abstract

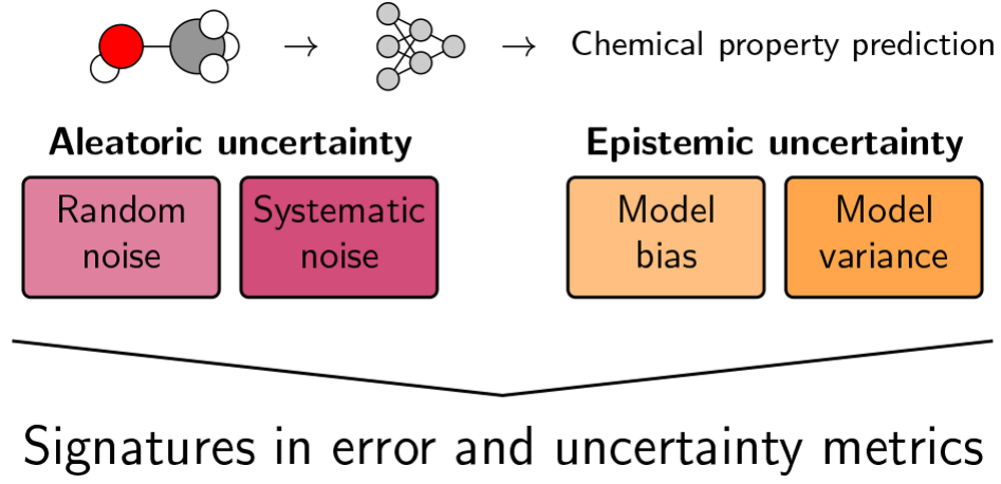

Characterizing uncertainty in machine learning models has recently gained interest in the context of machine learning reliability, robustness, safety, and active learning. Here, we separate the total uncertainty into contributions from noise in the data (aleatoric) and shortcomings of the model (epistemic), further dividing epistemic uncertainty into model bias and variance contributions. We systematically address the influence of noise, model bias, and model variance in the context of chemical property predictions, where the diverse nature of target properties and the vast chemical chemical space give rise to many different distinct sources of prediction error. We demonstrate that different sources of error can each be significant in different contexts and must be individually addressed during model development. Through controlled experiments on data sets of molecular properties, we show important trends in model performance associated with the level of noise in the data set, size of the data set, model architecture, molecule representation, ensemble size, and data set splitting. In particular, we show that 1) noise in the test set can limit a model’s observed performance when the actual performance is much better, 2) using size-extensive model aggregation structures is crucial for extensive property prediction, and 3) ensembling is a reliable tool for uncertainty quantification and improvement specifically for the contribution of model variance. We develop general guidelines on how to improve an underperforming model when falling into different uncertainty contexts.

## Introduction

Machine learning models for chemical applications such as predicting molecular and reaction properties are becoming not only increasingly popular but also increasingly accurate, for example for quantum-mechanical properties,^1−3^ biological effects,^4−6^ physicochemical properties,^7−11^ reaction yields,^12−14^ or reaction rates and barriers.^15−19^ Also, promising developments in the fields of retrosynthesis^20−24^ and forward reaction prediction^25−28^ have been made.

However, despite the increase in accuracy, many machine learning models fail in real-world applications.^29,30^ This can be due to a lack of generalization, lack of ability to filter out erroneous predictions for edge cases, or because the employed training and test sets are simply not reflective of the application of interest, so that the developed model is suboptimal for the proposed task. Poor choice of a test set can overestimate or, more commonly, underestimate the actual errors that a user will encounter when the model is applied. Optimizing a mediocre model can be tedious, time-consuming, and often unfruitful. Moreover, the model architectures, input representations, and data set characteristics for chemical applications differ considerably from other fields of research, so that following general guidelines for optimizing machine learning models often fails to produce accurate models for molecular and reaction properties. To optimize a model in a targeted and efficient manner, it is imperative to understand and identify possible sources of error and uncertainty in a model.

The separation of the total uncertainty into aleatoric (data-dependent, noise-induced, irreducible) and epistemic (model-dependent, reducible) contributions^31^ has recently received increasing attention.^32−34^ The aleatoric uncertainty is often referred to as the irreducible component of uncertainty that cannot be overcome by improvements to the model. Reduction in aleatoric uncertainty can instead come from improvements in the data itself, such as adding repeat measurements or removing erroneous entries. In contrast, epistemic uncertainty characterizes the reducible uncertainty caused by missing knowledge and can be decreased as the model is improved.^35^ The epistemic uncertainty can further be split into uncertainty arising from the choice of model (architecture, representation, and featurization) and the ambiguity of parameter optimization once a model is chosen.^35^ In this work, we follow the convention^36,37^ of calling the former model bias and the latter variance, but different other names are sometimes used in the literature, such as model uncertainty and approximation uncertainty.^35^ The difference between reducible and irreducible uncertainty can become blurred in these considerations, especially for different model architectures, different representations, and different data and test sets.^32,35^ Small data set sizes not only contribute to both bias and variance components of epistemic uncertainty because they cause some ambiguity in the optimal model parameters due to sparsity in some regions but also hinder the model convergence to a meaningful minimum generally. The size or nature of the data may additionally influence the choice of model architecture or machine learning method, providing a further avenue by which aspects of the data can feed into epistemic uncertainty.

Many approaches toward characterizing the uncertainty of a prediction exist, such as mean-variance estimation,^38^ Bayesian approaches,^39^ ensembling,^40−43^ evidential learning,^44^ and conformal predictions,^45^ among many others.^35,46,47^ Most approaches tackle aleatoric uncertainty, as well as those parts of the epistemic uncertainty that are associated with the ambiguity of the model parameters. However, uncertainty from model bias is usually omitted.^35^ Even when the aleatoric error is low and plenty of data is available for training, model bias can still prove to be significant. Model bias can have many forms and causes, among them limited flexibility of the model, limited data coverage, incomplete feature representation of the input data, poor training convergence to an appropriate model, and poor generalizability of training to the test set or to actual applications. We discuss how, especially in chemical systems, uncertainty from model bias can be a large contribution toward the error in a model’s prediction.

Despite the many works on characterizing uncertainty, little advice exists on how to optimize a suboptimal model once the sources of uncertainty are known. Furthermore, the circumstances under which the epistemic uncertainty modeled by ensembling is actually indicative of the true error are not well researched yet, despite its popularity.^46^ We therefore studied the performance of selected deep learning models on chemical regression prediction tasks where we systematically vary noise in the input data, the number of data points, the chosen model architecture, molecular representation, and the number of models in an ensemble. To this aim, we not only rely on literature data sets but also construct a new, noise-free, chemical data set. In the discussion, we then put forward general guidelines for how to detect and circumvent model errors caused by noise, bias, and variance. We pay particular attention to predictions of physicochemical targets, since we find some of the sources of uncertainty to be specific to chemistry.

## Methods

### Data Sets

In this work, a synthetic data set was constructed for molecular enthalpy at 298 K in units of kcal/mol as calculated from group additivity coefficients, based on the Benson group-increment theory.^48^ The data set was desired to have characteristics well-suited to the analysis of errors of different types: no inherent noise, large data set size, and a property function that was fully described by the features available to the model. With these characteristics, a model trained on the data set could be driven to extremely low levels of noise, bias, and variance error. By starting from a data set where very low error levels of all types are possible, the data set can then be manipulated to elevate errors in a controlled way to exemplify situations in which model performance is dominated by the different types of error.

We were motivated to generate a synthetic data set for this study due to the lack of noise-free data set options. Data sets generated by density functional theory (DFT) calculation are often considered for the role of a low-noise chemical data set as they are not subject to experimental uncertainty in data collection like most data sets would be. Indeed, DFT data sets are available and with large data set sizes in the case of QM9^49^ or PCQM4Mv2.^50,51^ The properties calculated in these data sets depend on the 3D atomic coordinates used in the calculation. The choice of a different molecular conformer to be used in calculation or a different optimization process to find the optimized atomic coordinates would result in different property targets. In our study of error types, we are using the connectivity graph representations of molecules, commonly referred to as 2D representations. The models using 2D graph representations do not distinguish between variations in optimized 3D coordinates or choice of molecular conformer, meaning that the models would not have access to all the features necessary to calculate the property and therefore would have some level of irreducible error, manifesting as noise. By creating a group-additivity data set, the features necessary to calculate the modeled property are explicitly available with a 2D model representation, allowing us to avoid this source of irreducible error.

The synthetic group additivity data set was constructed using publicly available data and molecules. Group additivity coefficients were fitted to the enthalpies calculated for the 134 thousand molecules of the QM9 data set^49^ using ridge regression. Group structures were defined by a central non-hydrogen atom and the atoms and bonds within a 1-bond radius. Only groups that were represented at least 100 times in QM9 and molecules made up entirely of those groups were included in the regression. The group additivity coefficients were rounded to the nearest thousandth kcal/mol. No non-nearest-neighbor group contributions were included for symmetry, ring strain, or other inter- or intramolecular interactions. The fitted coefficients were then applied to a larger set of molecules, the GDB11 set of over 26 million unique molecules containing up to 11 C/N/O/F atoms.^52,53^ Choosing only molecules made up entirely of structures included in the fitted coefficients, we obtain 7.9 million molecules. The result is a large data set with a property function that can be exactly calculated and relies on the local graph structure of molecules. Because the groups are only defined in terms of local connectivity, we expect the directed message passing encoding used by our model will be able to fully learn the necessary representation. The only inherent noise is at the level of numerical precision. Accurate representation of experimentally observable enthalpies was not a consideration in the construction of this data set and would be unnecessary for it to be used in evaluating contributions of different error types. This data set and models trained from it should not be used for estimation of experimental molecular enthalpies. Our data set of artificial enthalpy values is available for public download from a Zenodo repository.^54^

Models trained on the group additivity data set were evaluated using a single held out test set comprising 10% of the data set (790,681 data points), chosen randomly. When the number of data points used in training are indicated in figures, that is the combined number of data points in the training and validation sets, split randomly at a ratio of 80:20. When multiple submodels are combined in an ensemble for the synthetic data set, the same data splits are used in each submodel. Ensemble submodels are differentiated by beginning training of the model from different random parameter initializations. When the number of data points used for training is unspecified, a consistent set of about 0.7 million data points is used, corresponding to 10% of the nontest data remaining in the data set.

Furthermore, the QM9 data set^49,55^ was used as a low-noise real-world data set. We selected the enthalpy at 298 K and internal energy at 0 and 298 K as size-extensive properties, as well as the HOMO–LUMO gap as a size-intensive property. We trained directly on the quantum chemical energies, without subtraction of the atomic reference values. A single held out test set comprising 10% of the data (13,083 data points) was used. The rest of the data was used for the training and validation sets, where a specified number of data points were selected randomly and split into training and validation sets in ratios of 80:20. To compute learning curves using QM9, i.e. the model performance dependent on data set size, differently sized training and validation sets were drawn containing a specified number of data points *N*, while leaving the test set untouched. Submodels in an ensemble using the QM9 data share the same data splits with different initial model parameters.

### Model Structure

Three machine learning architectures were employed within this study: (i) directed message passing neural networks (d-MPNNs) as described by Yang et al.^8^ and implemented in the Chemprop software package^56^ as a class of 2-dimensional graph-convolutional neural networks using learned representations, (ii) feed-forward neural networks (FFNN) on molecular fingerprints, and (iii) the 3-dimensional convolutional neural network SchNet.^57^ Ensembles of five models were trained for each architecture and task if not specified otherwise.

The d-MPNN model takes the molecular graph as input and performs several steps of message passing to update atom and bond features with information from their neighborhood to yield an atomic representation. A molecular representation is then obtained by aggregating the atomic representations using an aggregation function such as summing or averaging. Subsequently, a feed-forward neural network transforms the learned molecular representation into the respective target property. Unless otherwise noted, d-MPNN models trained on the synthetic group additivity data set use a hidden size of 1000, four steps of message passing, two feed-forward layers, scaled sum aggregation (called “norm” in Chemprop), and 200 epochs of training unless otherwise indicated. In contrast, d-MPNN models trained on QM9 differ slightly by using a hidden size of 300. In the following, the hidden size is specified, that is the hidden size in both the d-MPNN and the FFNN parts. All other hyperparameters were chosen according to their default values in Chemprop.

FFNNs take a molecular fingerprint, here a Morgan fingerprint^58^ as implemented in RDKit,^59^ as input and transform it into the respective target property. We used FFNNs as implemented in ref (56), where we omitted the message-passing. Model training used a hidden size of 300, two feed-forward layers, and 200 epochs of training unless indicated otherwise.

SchNet was used as provided in ref (57) with default hyperparameters and trained only on QM9 tasks. It takes as input the nuclear charges and coordinates of each atom in a molecule which are calculated using quantum chemistry and provided along with the QM9 data set. The atomic representations of each atom are refined using continuous-filter convolutional layers, thus taking into account other atoms in the molecule based on their relative distance. The atomic representations are then utilized to compute atomic contributions to the overall target, which are subsequently averaged or summed up to the total molecular target value. Since SchNet does not directly support ensembling, models with different initialization seeds were trained manually, and their predictions were averaged for each data point in the test set.

For all architectures, the validation set was used to select the best model within 200 epochs, which was further used to evaluate the test performance.

### Ensemble Metrics

Throughout this work, we refer to the predictions made by and errors resulting from ensembles of submodels. To explain the meaning of different ensemble metrics, we will use *ŷ*_*i,j*_(*X*_*n*_) to denote the model prediction on the input *X*_*n*_ (test data molecule *n*), where *i* indicates model initialization, and *j* indicates the split configuration of the full data into training, validation, and test sets. The target for each data point is given by *y*(*X*_*n*_). For ensembling, we obtain *N*_ens_ models with different *i* on the exact same data splits *j* = 1. The prediction of the ensemble, *y* (*X*_*n*_), is given by
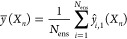
1where the submodel predictions for a particular test data point *n* are averaged together over the number of submodels included in the ensemble, *N*_ens_. The reported mean absolute error of an ensemble model is
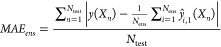
2(with an analogous expression for the root mean squared error). Here, we find the absolute error between the ensemble prediction and its corresponding target value. The overall model performance is reported as an average over all *N*_test_ data points in the test set.

The standard deviation of the ensemble prediction of each point *n* may be used to define confidence intervals and uncertainty bounds. The standard deviation used is the unbiased standard deviation of the submodel predictions for each data point.
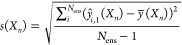
3

The standard deviation of the ensemble prediction is used to define uncertainty intervals in two different ways in this work. In the case where we use the measure directly to evaluate error magnitude (Figure 8), we will define the confidence interval for predictions of each test data point *n* indicated by the standard deviation as
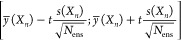
4where *t* is the Student-t factor for the specified confidence *p* and degrees of freedom *N*_ens_–1. In the case where we are using the ensemble standard deviation only as a relative indicator of total error within the data set (Figure 3), the uncertainty bounds will be the standard deviation as given in eq 3 and scaled to match the average error of the competing uncertainty method.

The ensemble mean and standard deviation *y* (*X*_*n*_) and *s*(*X*_*n*_) can further be used to estimate the contributions of bias and variance error to the overall observed MAE via Bayesian inference. Here, we follow the method of ref (60). In this approach, the different predictions made by individual models *i* for a single test data point, *ŷ*_*i,j*_(*X*_*n*_), are assumed to be normally distributed around a mean distribution value μ_*j*_(*X*_*n*_), with a spread related to the ensemble standard deviation *s*(*X*_*n*_). In accordance to the central limit theorem, an ensemble prediction *y* (*X*_*n*_) will converge to μ_*j*_(*X*_*n*_) at very large ensemble sizes. The nonvariance contribution to error is considered to be the absolute error occurring in a theoretical very large ensemble
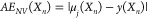
5The nonvariance error consists of bias and noise errors, and in noise-free data sets it represents only the bias error. The variance error is considered to be the difference between the total absolute error of the ensemble prediction and the nonvariance error
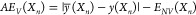
6Bayesian inference is used to calculate the posterior distribution of μ_*i*_(*X*_*n*_) – *y*(*X*_*n*_) for each data point, using the distribution of *y*(*X*_*n*_) – *y*(*X*_*n*_) over the data set as an initial prior distribution, which is subsequently iteratively refined. The posterior distribution can be used to calculate expected values of the absolute error from variance and nonvariance defined in eq 5 and eq 6 for each data point. The contributions are then averaged across the data set to arrive at the expected variance and nonvariance contributions to the data set MAE.

### Software and Data Availability

The Chemprop software^56^ and SchNet software^61^ used in model training are both freely available through GitHub. The constructed noise-free data set of group additivity enthalpies is available through Zenodo.^54^ The QM9 data set can be downloaded from the MoleculeNet Web site.^9^ The implementation of the Bayesian inference method for calculating nonvariance contribution is available through GitHub (https://github.com/cjmcgill/ensemble_projection).^62^ Other scripts necessary to train the models analyzed in this work and recreate the results are provided through GitHub (https://github.com/cjmcgill/characterizing_uncertainty_scripts).^63^

## Results

In the following, we describe the influence of noise, bias, and variance on the observed model performance, as well as possible pitfalls associated with each type of error. We often discuss the shape of the learning curve, i.e. the test set error as it depends on the size of a data set, as different types of limitations caused by noise, bias, or variance can lead to unique patterns in the learning curve. The slope of the learning curve characterizes the change in error upon addition of data and can be utilized to predict how much data is needed to achieve a specific accuracy. In general, a steep, negative slope on a log–log plot without plateaus is desirable.

### Noise

Noise in the target data obstructs a model’s ability to learn meaningful relations between an input and a target. In general, noise can be of random, uniform nature (homoscedastic), afflicting all data points with the same error probability distribution, or systematic (heteroscedastic), where different domains of data are affected by different error probability distribution. We discuss both options separately in the following, because they require different remedies. In our demonstration of random noise, we also show that noise has distinct effect behaviors when it is present in the training set versus the test set, with the effects in the training set actually leading to reducible errors that can be improved with additional training data, whereas the effect of noise in the test data is irreducible.

#### Random Noise

**Figure 1:**
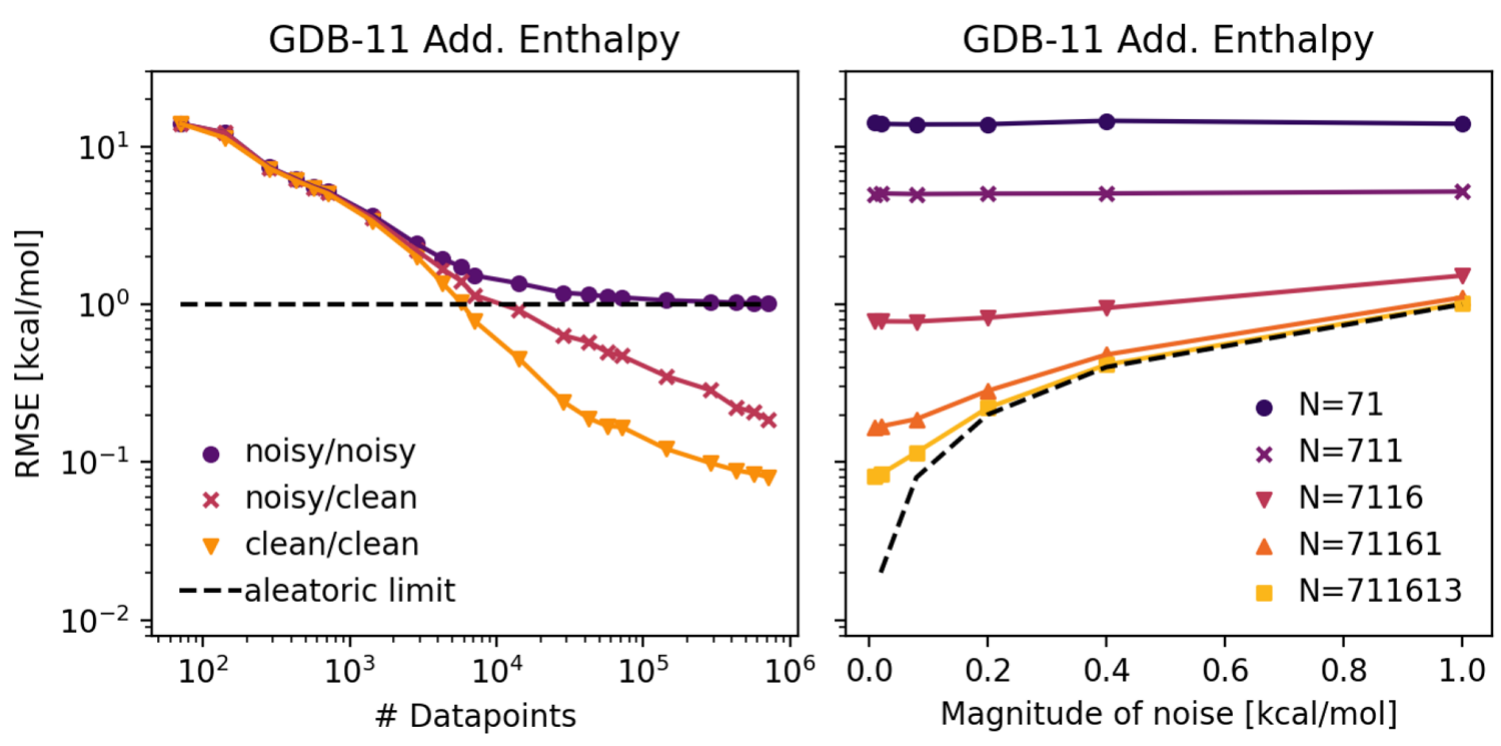
Left: Influence of random noise (magnitude of 1 kcal/mol) in the training and test sets on the reported root mean squared error of the test set as a function of the data set size. The labels indicate whether noise was applied to the training/test sets. Right: Dependence of performance on the magnitude of noise in the case where noise was applied to both the test and training sets. The black dashed line describes the aleatoric limit, where the observed RMSE equals the standard deviation of the noise distribution. The labels indicate the size of the data set used for training (*N*). Noise is applied to both the training and test sets.

To showcase the influence of noise on a machine learning model, we use the noise-free data set of artifical, additive enthalpies to train a d-MPNN model. The respective model performance with different sizes of the data set is depicted in Figure 1 for different levels of noise. For clean, noise-free training, validation, and test sets (labeled “clean/clean”, left panel), a standard d-MPNN can learn the target property to seemingly arbitrary accuracy, because the task is simple and learnable. Adding Gaussian noise with standard deviation, i.e. magnitude, of 1 kcal/mol to the training data but not the test data (labeled “noisy/clean”, left panel) leads to a loss in performance, diverging after the RMSE for the clean model approaches the noise level. The model continues to learn with added data and could still achieve reasonable accuracies, requiring more data for the same performance compared to the model trained on the clean data. Though noise-based, the error from noise introduced while training is not irreducible. However, when noise also affects the test set (labeled “noisy/noisy”, left panel), it leads to an additional perceived loss in observed performance. The trained model is the exact same for the “noisy/clean” and “noisy/noisy” curves; only the test set differs in the addition of noise. The true model performance is thus described by the “noisy/clean” curve, but instead the noise in the test set causes the “noisy/noisy” curve to be observed. The learning curve of the noisy test and training sets approaches an asymptote at 1 kcal/mol, which is the standard deviation of the employed noise distribution. Upon addition of more data, no further improvement in observed performance is perceived. The aleatoric limit is reached, where the observed test set error is dominated by noise. The effect of noise in the test set on the perceived model error is irreducible. This aleatoric limit is not a true limit of the model performance, however, but a property of the test set used to evaluate the model. Users who observe this sort of asymptotic behavior with respect to the data set size should consider test set noise as a possible cause.

The right panel of Figure 1 depicts the observed test set performance of noisy test and training sets with different levels of noise and different numbers of training points. We can see how the model performance changes as it approaches the aleatoric limit (dashed black line) where the RMSE equals the standard deviation of the noise distribution. With a small number of training points, such as 71 or 711 (indigo and violet curves), the test set error is not governed by noise (but instead dominated by bias and variance errors caused by the tiny number of data points), so that the magnitude of added random noise does not influence the observed performance significantly. As the aleatoric limit increases and approaches the performance of the other three data set sizes, the RMSE of the data sets is deflected upward. As the noise level surpasses the baseline non-noise error for the data set sizes, model performances converge and become indistinguishable as can be seen at the 1 kcal/mol noise level for the two largest data set sizes. A similar trend with the presence of an aleatoric limit due to controlled addition of noise was also noted by Xie et al.^64^

**Figure 2:**
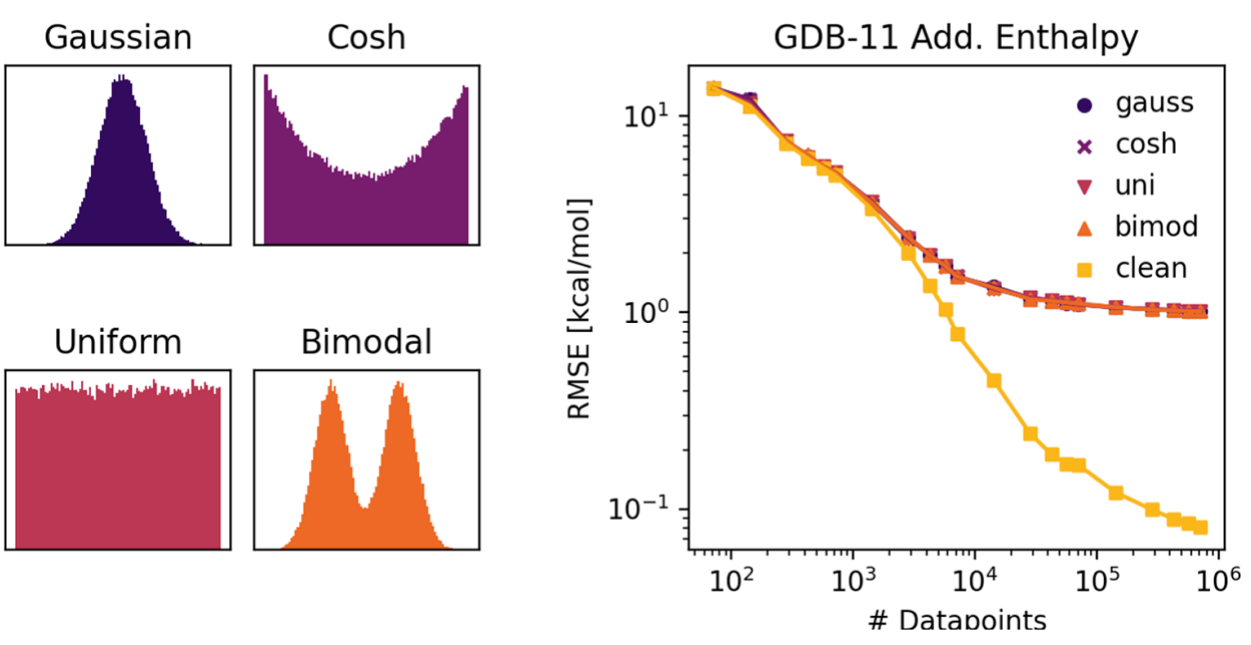
Performance for models trained with different noise distributions applied to the data set. Both the training and test sets contain noise. Left: The applied noise distributions, each with standard deviation of 1 kcal/mol, shown at left. Right: Root mean squared error for the different noise distributions as a function of data set size. The four noisy data sets yield very similar performance (points overlap in the figure).

The noise we discussed so far was drawn from a Gaussian distribution. We also tested uniform, hyperbolic, and bimodal noise distributions, where the respective parameters were chosen so that each distribution had a standard deviation of 1 kcal/mol and was centered around 0 kcal/mol. Figure 2 depicts the respective distributions and their observed model performances. Both the training and test sets contained noise. We did not observe any difference in overall model performance between different error distributions, as long as the mean and standard deviation of the noise was the same, respectively. Though noise distributions found in real data may be non-Gaussian, if homoscedastic, they should still follow the same trends of approaching an asymptote due to noise.

#### Systematic Noise

If different regions of chemical space lead to larger noise levels, it is possible for a model to learn which regions are unreliable if the loss function is adapted accordingly, as first reported by Nix et al.^38^ When a model is trained using mean-variance estimation, the model outputs two values per target instead of one, namely the mean and variance. The two outputs of a mean-variance estimation model describe the model prediction probabilistically, with the mean being the center of the prediction distribution and the variance indicating the Gaussian spread of uncertainty around the mean. Other variations and extensions of mean-variance estimation also exist, such as evidential deep learning where the values returned by the model express uncertainty distributions for the values of the mean and variance.^44^ Mean-variance estimation and similar techniques can be very successful in training models on noisy data sets if the error is a function of the input features, since it allows the model to learn on which data points to focus and which to regard as unreliable.^34,46,65−67^ However, it is not amenable to noise that is uniformly distributed over all data points or systematic noise that is applied based on external factors not represented in the input features of the training data. For example, if one measurement instrument had increased noise in data collection but the identity of the instrument used in collection was not included in the input features and could not be inferred from the input features, then the systematic noise applied according to the external factor of a faulty instrument would not be distinguishable. Concerns around suboptimal performance of mean-variance estimation techniques have been recently reported in the literature.^67^ We therefore recommend that users consider whether there are identifiable sources of systematic noise related to model input features and that they compare performance of a mean-variance estimation model against a simple model.

**Figure 3:**
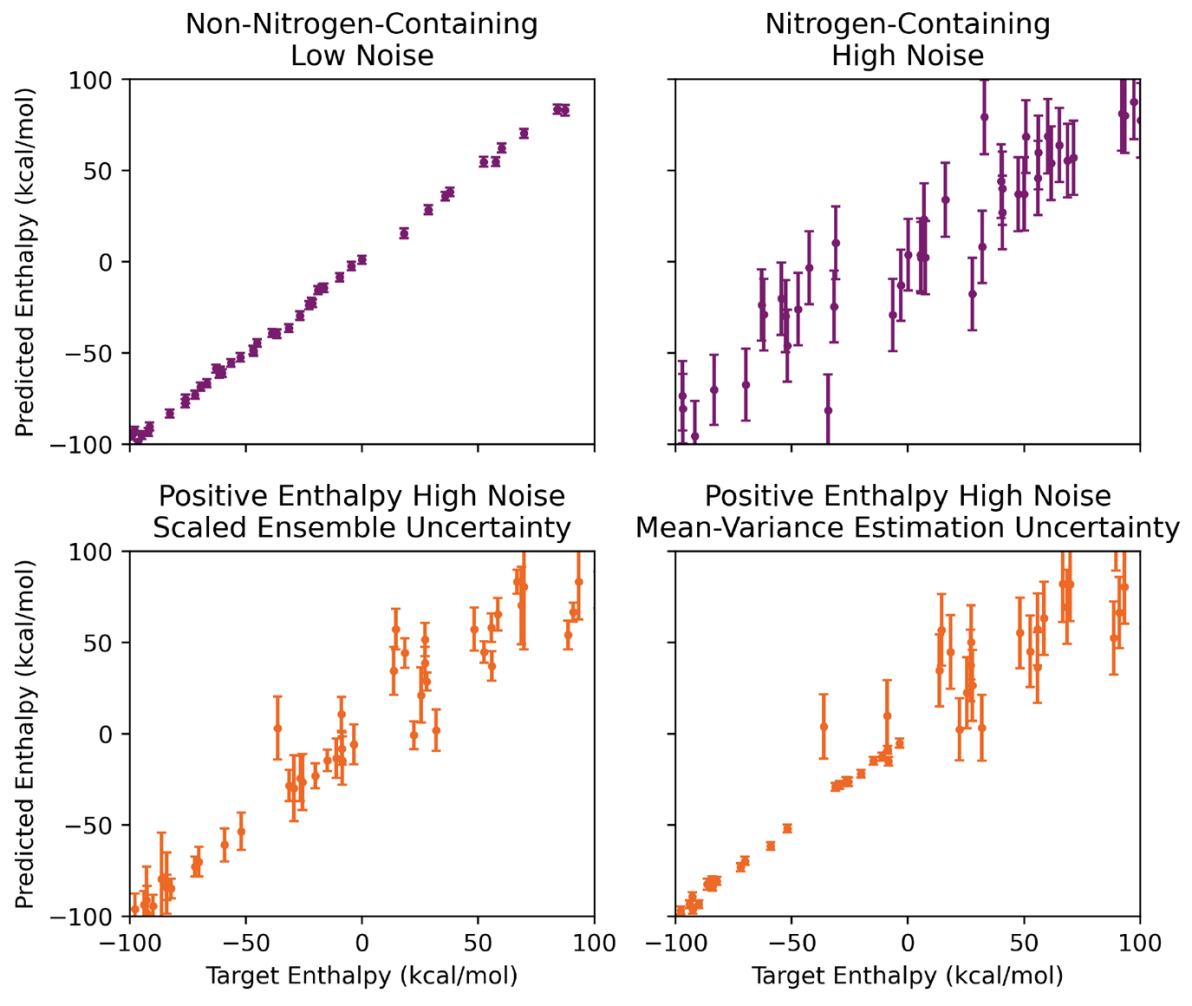
Examples of uncertainty methods being used in models with distinct systematic noise regimes. One model (violet) was trained and evaluated with 20 kcal/mol standard deviation noise applied to nitrogen-containing molecules and 2 kcal/mol standard deviation for non-nitrogen-containing molecules. The mean-variance estimation method is able to quantitatively distinguish between the low noise (top left) and high noise (top right) regimes. A second model (orange) was trained and evaluated with 20 kcal/mol standard deviation noise applied to positive enthalpy molecules and 2 kcal/mol standard deviation noise applied to negative enthalpy molecules. The ensemble variance method (bottom left) is less able to distinguish the noise regimes than mean-variance estimation (bottom right).

As with our demonstrations of behavior under random noise (Figure 1, Figure 2), we use the data set of noise-free additive enthalpies to demonstrate behavior under systematic noise. We use two different cases of systematic noise application as demonstrations, using training data set sizes of 711,613 data points. In the first case (Figure 3, violet), we apply Gaussian noise of standard deviation 20 kcal/mol for nitrogen-containing molecules and Gaussian noise of standard deviation 2 kcal/mol for non-nitrogen-containing molecules. When training a model to predict these data points using a mean-variance estimation approach, the model is able to distinguish between the noise regimes of the non-nitrogen-containing molecules and the nitrogen-containing molecules. For non-nitrogen-containing molecules in the test set, the model has an RMSE of 2.12 kcal/mol and a mean predicted standard deviation of 2.35 kcal/mol. For the nitrogen-containing molecules in the test set, the model has an RMSE of 20.0 kcal/mol and a mean predicted standard deviation of 20.0 kcal/mol. We see that in this case where the noise is the predominant error source and clearly delineated based off the input features to the model, the mean-variance estimation method performs well at quantifying the error magnitude in the different noise regimes.

In the second case (Figure 3, orange), we apply a Gaussian noise of standard deviation 20 kcal/mol for molecules with positive enthalpy and Gaussian noise of standard deviation 2 kcal/mol for molecules with negative enthalpy. For this case, we contrast the performance of uncertainty estimation by ensembling (bottom left) with the mean-variance estimation method (bottom right). As we discuss in a later section, ensembling is a measurement of variance error and does not directly incorporate noise error. Ensembling also requires a scaling calibration to match the magnitude of errors unless variance error dominates, so the ensemble uncertainty was scaled so that the ensemble and mean-variance estimation would have the same average value. In this case, the ensembling method of uncertainty estimation does a poor job of distinguishing the noise regimes, with a mean uncertainty of 10.6 kcal/mol for molecules with negative original target enthalpy and a mean uncertainty of 11.5 kcal/mol for molecules with positive original target enthalpy. The mean-variance estimation is able to distinguish and quantify the noise regimes appropriately, with a mean uncertainty of 2.4 kcal/mol for molecules with negative original target enthalpy and a mean uncertainty of 19.5 kcal/mol for molecules with positive original target enthalpy. This example shows how mean-variance estimation can distinguish between noise regimes better than a method suited to other error types.

### Bias

For noiseless data sets, the accuracy of a model in general increases with the size of a data set, as visible and discussed in Figure 1. The performance is also influenced by the model size, i.e. the number of parameters, as well the input representation and architecture of the model. These factors contribute to the error caused by model bias and are discussed in the following.

#### Data Coverage

**Figure 4:**
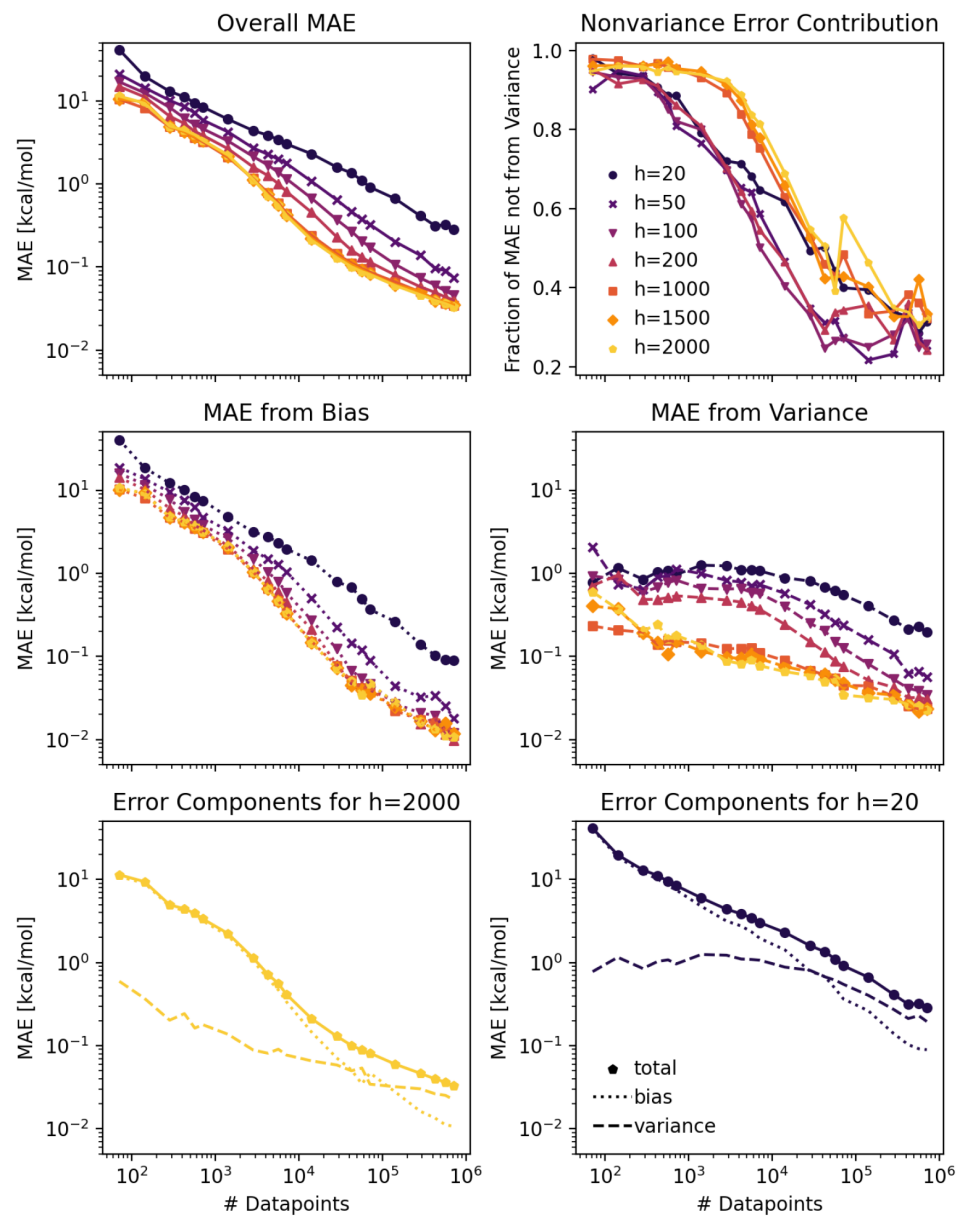
Top left: Mean absolute errors as a function of the data set size for different model sizes. Top right: Fraction of the reported test set error not originating from the variance error. Middle left: The mean absolute error attributable to the bias error as a function of the data set size for different model sizes. Middle right: The mean absolute error attributable to the variance error as a function of the data set size for different model sizes. Bottom: Contributions to the total error from variance and bias to the performance with a hidden size h = 2000 (left) and h = 20 (right). Here, bias accounts for all nonvariance error.

We first discuss model bias errors due to the number of data points, using models trained with the d-MPNN. Figure 4, top left, depicts the model performance as a function of the training set size and model size (size of hidden layers in the message passing and feed-forward networks). For a model of a given number of parameters, increasing the number of data points increases the accuracy of the model’s predictions, where the slope on a log–log plot is nearly independent of the number of parameters. The error reduction with more data is presumptively the data coverage error, but where is this data coverage error coming from? Is it caused by model bias, where a low number of data points does not allow the model to find the true global minimum in the high-dimensional parameter space? Or is it caused by variance, where differently initialized models converge to a distribution of model outcomes with associated random variations in observed error? Our recent work applying Bayesian inference to ensembling uses the observed variation in prediction error within an ensemble to estimate the distribution of errors before variance is applied, i.e. the nonvariance component of the error.^60^ Using this method, we decompose the total error into contributions from variance and bias (here, bias is computed as all error not from variance, which is a valid assumption for a noiseless data set). The mean absolute errors shown in Figure 4 are for single models, though the inference of the bias and variance contributions was made using the distribution of predictions observed in ensembles of 5 submodels.

The middle left panel of Figure 4 shows the trends in the bias error across data set sizes and for different model sizes. These learning curves show that in these demonstrations, increasing the number of training data reduces the bias error present at a rate that is roughly linear on a log–log plot. Unsurprisingly, the bias error is most severe when the training data set size is small.

The middle right panel of Figure 4 shows how the variance error decreases with the data set size as well. The slope of the decrease is steeper with the bias error than with the variance error. This dynamic gives rise to the changing proportion of error attributable to bias (top right panel). At small data set sizes, the error is dominated by bias errors with only a small fraction due to variance. As the data set size increases, both the bias and variance errors decrease, but the proportion of error steadily becomes dominated by the variance error. The bottom panels of Figure 4 depicts the total error for hidden sizes of 2000 (left) and 20 (right) decomposed into variance (dashed line) and bias (dotted line), showing the transition from the bias dominated error to the variance dominated error clearly in the two extreme model size cases.

In this case, adding data is an important factor to decrease model bias and arrive at a model that is mainly limited by errors stemming from variance. In general, the vast chemical space makes data size and coverage a large source of error compared to other fields of research, where many chemical structures are unique or under-represented in (experimental) data sets. The implications of this shortcoming on uncertainty estimations are discussed later in this work. In the following, we first investigate other possible sources of model bias.

#### Model Architecture and Representation

As visible in Figure 4, top left, the model performs better for a higher number of parameters for a given data set size, but the effect levels off, so that adding more parameters indefinitely is not advisible. A comparison of the bias errors shown in Figure 4, middle left panel, shows that increasing the number of parameters decreases the absolute magnitude of the error from model bias. A steady improvement in the bias error appears to be present across all data set sizes as the model hidden size is increased from 50 to 1000, though further improvement with increases to hidden sizes of 1500 and 2000 are not readily apparent. A too small model (for example *h* = 20) therefore contributes to model bias and should be avoided.

Besides the model size, there are also other factors contributing to model bias, such as molecular representations and model architectures. We explore the effects of architecture and representation by comparing the performance of a d-MPNN to SchNet. Message passing neural networks are built on 2-dimensional graph representations, whereas SchNet takes the 3-dimensional coordinates as input. We therefore expect SchNet to perform better for targets that depend on the 3-dimensional conformation, such as the enthalpy in the QM9 data set.

**Figure 5:**
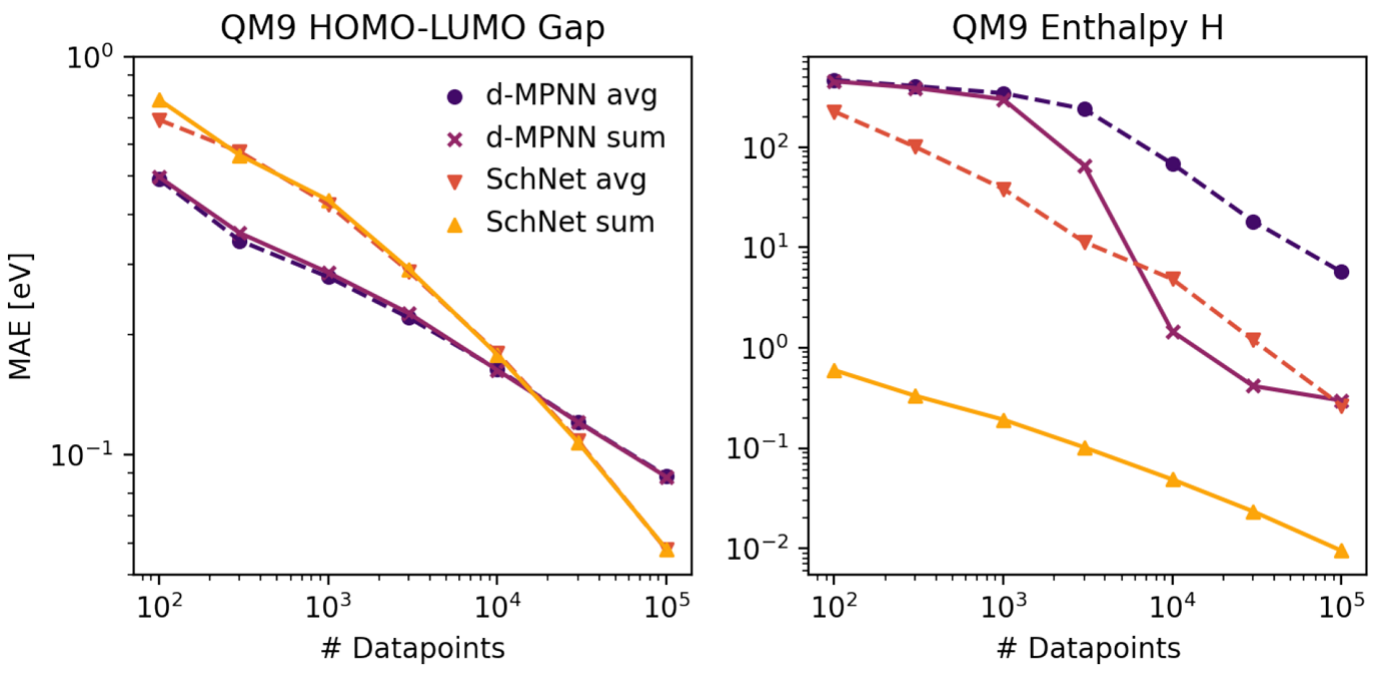
Mean absolute errors as a function of the data set size for mean (dashed line) and sum (continuous line) aggregation for d-MPNN (2D) and SchNet (3D) models. Left: Enthalpies H(298 K) from QM9. Right: HOMO–LUMO gaps from QM9.

The left panel of Figure 5 depicts the mean absolute errors of a d-MPNN and SchNet for HOMO–LUMO gaps. The 3-D method (SchNet) needs more training data to perform well than the simpler 2-D method (d-MPNN) but provides better performance with very large data set sizes. To choose the best model for a given data set, it is therefore advisible to take into account the size and diversity of the data. For small (or highly diverse and sparse) data sets, a simpler model is often preferred.

Besides the general model architecture, many smaller details and hyperparameters largely influence model performance, too. We showcase this effect by examining the influence of the aggregation function that combines atomic into molecular representations or properties. Both d-MPNN and SchNet first compute vectors of properties for each atom in a molecule and then combine these atom vectors to construct a single fixed-length learned-fingerprint vector for the molecule. This vector is the input to a conventional feed-forward neural-net in the d-MPNN or directly produces the target within SchNet. However, what is the best way to combine the atom vectors, by averaging or summing? In Figure 5 (left), one can see that either method of combination works about equally well for predicting HOMO–LUMO gaps. But in Figure 5 (right), the “sum” method works much better than the “avg” method. This is because enthalpy is an extensive quantity, that increases more or less linearly with the number of atoms. If one averages (rather than sums) the atom vectors, one loses information about how many atoms are in the molecule. In contrast, the HOMO–LUMO gap has a much weaker dependence on molecular size, it is more like an intensive quantity, so it can be modeled using “avg” about as well as it is modeled using “sum”. An extensive (size-conserving) representation and architecture is therefore essential for size-extensive properties like the energy.^68^ However, it can be easily overlooked when training models, especially when training multitask models for a mixed set of extensive and intensive targets such as the QM9 data set which contains both. As visible in Figure 5, choosing an intensive architecture (averaging over all atoms) for an extensive property such as the enthalpy leads to large performance losses for both the d-MPNN and SchNet. For an intensive property, there is nearly no difference in performance, so we recommend using extensive representations and architectures when in doubt.

In Figure 5 (right), we furthermore observe that the enthalpy which largely depends on the 3-dimensional conformation can be modeled by a 3-D approach in much greater detail. However, a direct comparison is difficult since SchNet differs not only in the general architecture but also in the way the model is initialized. Namely, SchNet utilizes the mean and standard deviation of average atomic contributions to the target properties in the training set to initialize the model with a good guess of the target property of each molecule. This is especially helpful for extensive properties since it enforces additivity of the atomic contributions. As such, d-MPNN and SchNet are not directly comparable, since the d-MPNN has to explicitly learn the additivity from the data.

#### Featurization

**Figure 6:**
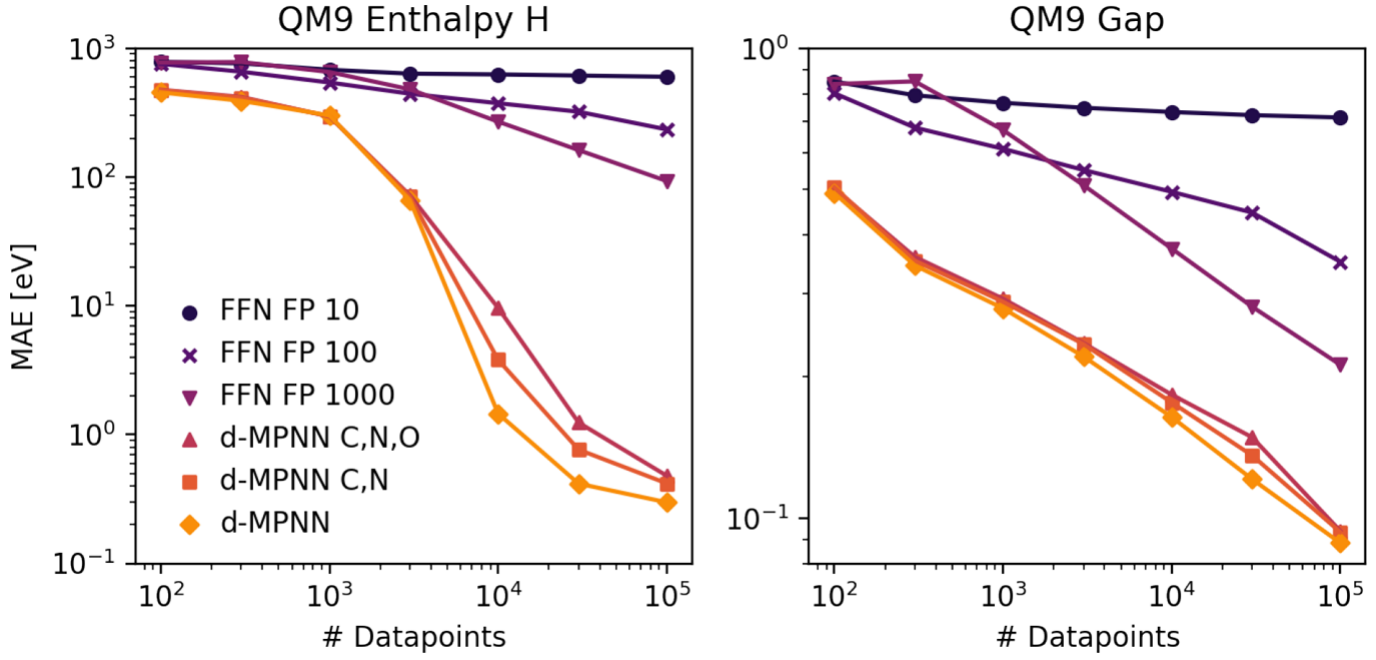
Mean absolute errors as a function of data set size for NN models with Morgan fingerprints with a radius of 2 and length of 10, 100, and 1000 as input compared to d-MPNN models with standard or disrupted atomic features (N represented as C in features or N and O both represented as C in features). Left: Enthalpies H(298 K) from QM9. Right: HOMO–LUMO gaps from QM9.

Once a model architecture and representation for molecules (2D graph, 3D coordinates, fingerprint, string) has been chosen, there are still many options for what input features to use for the encoding of molecules within that representation. The inclusion of features relevant to the target property can make a significant difference in the ability of the model to learn the property function.^68,69^ Errors due to the choice of input features are a form of model bias. Figure 6 depicts model performances for the QM9 targets enthalpy and HOMO–LUMO gap for different model inputs. First, we skipped the message passing step and used a Morgan fingerprint^58^ of size 10, 100, or 1000 as input to a feed-forward neural network. Second, we modified the default d-MPNN representation of the molecular graph not to discern between carbon and nitrogen (labeled ‘d-MPNN C,N’) or carbon, nitrogen, and oxygen (labeled ‘d-MPNN C,N,O’) to artificially create bad features. For both targets, d-MPNNs outperform fingerprints, where smaller fingerprints lead to even worse performance. Bad features in the d-MPNN again decrease model performance. With increasing data, models with corrupted features can regain performance, since bias from featurization can be a reducible error source if the missing information can be learned, e.g. from the structure of the rest of the molecule. Thus, finding optimal features is less important for large data sets but essential for medium-sized data sets. However, error from featurization can also be irreducible if the model loses important information that it cannot learn or infer. This is the case for the fingerprint of size 10 in Figure 6, which is too small to faithfully represent the diversity of molecules present in the data set. Despite these insights being rather expected, we find that often not enough attention is paid to featurization when building new models. For example, targets like the enthalpy might require additional features such as ring sizes, which are not default in e.g. the implementation of d-MPNNs we utilized in this work. In fact, adding a one-hot encoded ring size to atom and bond features increases the performance of our d-MPNN from mean absolute errors of 0.30 to 0.19 eV for *N* = 100,000. We also recently trained d-MPNNs to predict solute parameters, solvation free energies, enthalpies, or solubilities, where we found atom features specific to solvation such as the presence of H-bond donors or acceptors to be key to good model performance.^11,70^ For the prediction of molecular UV–vis absorption peaks, we furthermore found that the inclusion of a model prediction trained on low-fidelity data as an additional custom molecular feature within a high-fidelity model can be beneficial.^71^

### Variance

As detailed in the previous sections, reducing error from (nonsystematic) noise and bias is a tedious and manual process that requires expertise and knowledge of the problem at hand. In contrast, error from variance can be tackled with an easy and automated, though computationally intensive method: ensembling.

#### Bias-Variance Relationship in Noiseless Data Sets

We engage with ensembling as a tool of reducing the variance error later in this section. First, we analyze trends previously noted in the Bias section that apply to the variance error for indications of when ensembling may be more effective.

Figure 4 shows trends in the bias and variance errors when trained on the noiseless group additivity enthalpy data set. A first noteworthy trend in this data set is the relationship between the variance error and model size (middle right panel). In much the same way that increasing model size improved the bias error for all data set sizes, larger models appear to gradually improve variance performance for all data set sizes up to a point of diminishing returns. This is significant because it shows a case where adding additional parameters tightens the distribution behavior for the performance of individual model instances. Adding more randomly initialized parameters decreases the randomness of the outcome, presumably due to improved convergence dynamics in a larger model.

The figure shows that as the data set size is increased, both the bias error and variance error decrease steadily (middle left and middle right panels). The bias error starts at a higher level but decreases more steeply, leading to a changing proportion of error due to variance and nonvariance sources (top right panel). The result is that the bias error dominates at low data set sizes and the variance error dominates at high data set sizes, regardless of the model size. In this demonstration, variance error contributes roughly 5–10% of the total error at low data set sizes and 60–80% of the total error at high data set sizes. This indicates in large data sets with low noise, that ensembling has the potential to be highly effective because the variance errors that it can reduce are so significant. We also can see that in high bias regimes, such as for low data set sizes, the variance error to be corrected is present but small, making ensembling a less attractive measure. The data set used for this demonstration is a relatively simple one, so users should expect that the proportions and data set size needed to transition between regimes will differ accordingly.

The nature of the transition between the dominant error regimes is of interest as well (Figure 4 top right panel). Though all of the considered model sizes trend toward higher variance contribution at large data set sizes, the proportions do not track tightly together. Variance becomes a significant error source at an intermediate data set size for the smaller model sizes than for the larger model sizes. The reason behind this is more due to differences in variance error behavior rather than bias error behavior. If we exclude the behavior of the hidden size 20 model as an outlier, the bias error versus learning curves (middle left) are relatively tightly clustered, with the error varying by roughly a factor of 2 between hidden sizes 50 and 2000. The variance error learning curves (middle right) are much less tightly clustered, with the error varying by roughly a factor of 10 between hidden sizes 50 and 2000 at intermediate data set sizes. This trend implies a need to consider larger model sizes when used with intermediate data set sizes in order to avoid the onset of significant variance losses.

#### The Importance of Ensembling

**Figure 7:**
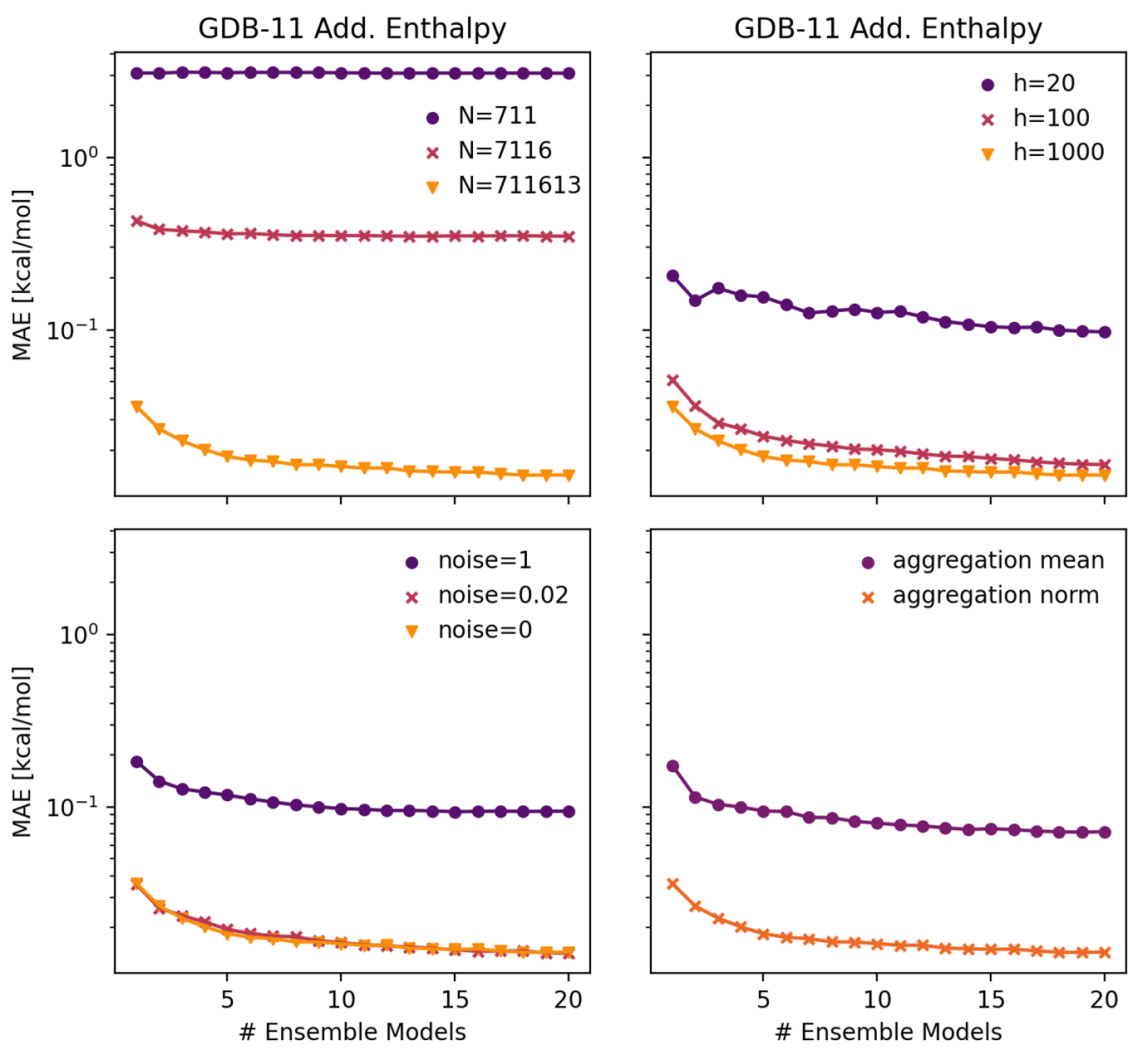
Mean absolute error as a function of ensemble size for different data set sizes (top left), different model sizes (top right), different magnitudes of noise in the training data (bottom left), and different featurization strategies (bottom right).

In this work, we produce ensembles of submodels by starting each training run from differently initialized model parameters. Many other techniques for generating randomly differentiated submodels exist, such as bootstrapping,^41^ Monte Carlo-dropout,^42^ or saving snapshots^43^ from different training epochs.^46,47^ Our reported model prediction is the average of the predictions of the submodels in the ensemble (eq 1). Figure 7 depicts the observed mean absolute error of the test set of several d-MPNN models trained on the artificial enthalpy data set as a function of the number of models in the ensemble. The different panels in Figure 7 refer to different situations in which additional model errors have been introduced with data coverage (top left), model size (top right), noise (bottom left), and architecture (bottom right). Regardless of the sources of additional error in the model, ensembling always improves model performance on average. The performance with increasingly large ensembles will approach an asymptote. This is because ensembling purely addresses variance error. A large ensemble can remove the variance error, as the ensemble prediction *y*(*X*_*n*_) will converge to the variance distribution mean μ_*j*_(*X*_*n*_) (eqs 5, 6). However, bias and noise errors remain even when an ensemble size is made very large. The lower the error from other sources, the larger the performance gains available from ensembling. This effect could already be anticipated from Figure 4, where a larger contribution of variance error was observed for lower data coverage error. We further note that the performance gains for adding an additional submodel to an ensemble are diminishing, while the computational cost of training and saving models scales roughly linearly with the number of submodels. Adding a small number of additional submodels to improve the model performance may be justified against the costs while adding a large number of additional submodels may not be.

#### Ensemble Variance as a Measure for Prediction Error

Training an ensemble of models and inspecting the variance between predictions of the individual submodels furthermore is a popular method to estimate the uncertainty associated with a prediction.^40,46,47,72,73^ Ensemble uncertainties can be used for risk management or active learning, among others, and are thus valuable information when judging the reliability of a prediction. However, uncertainties from ensembles only directly represent the true error for variance-dominated systems, i.e. the model uncertainty caused by model bias is not included. To showcase this, the deviation between the uncertainty from the ensemble variance and the true observed error was computed for all systems of Figure 7 using an ensemble of five submodels.

There are several available methods to evaluate uncertainty predictions that take into account different aspects of uncertainty. Here, we assess the quality of the uncertainty estimation by computing the calibration error curve, which is obtained by counting the fraction of test set data points that lie within a *p* confidence interval around the predicted value. Confidence intervals were modeled via eq 4 on a single split. For a perfectly calibrated model, the observed, empirical coverage (fraction of the test set with targets within each interval) should equal *p*, i.e. 95% of the test set should have a true target value within the 95% confidence interval spanned by the ensemble mean and variance of each prediction. The area under the calibration error curve, AUCE, measures the deviation of the observed calibration curve from perfect calibration. An AUCE of 0 corresponds to perfect calibration; larger values indicate an imperfect calibration.

**Figure 8:**
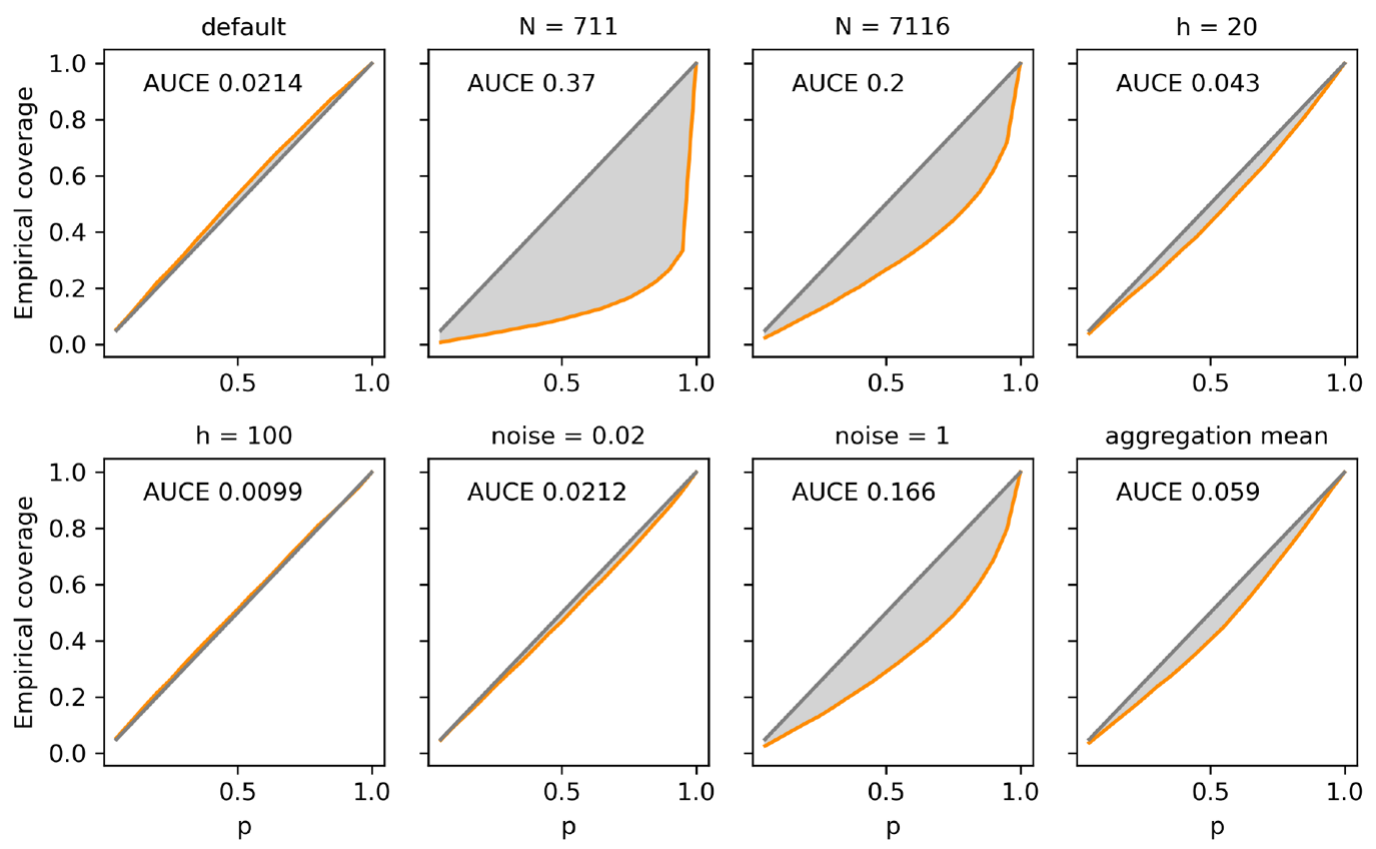
Confidence-based calibration curves (orange) for different models trained using the group additivity enthalpy data set. The area under the calibration error curve, AUCE, is highlighted in light gray; perfect calibration corresponds to the diagonal line in gray. If not specified otherwise, *N* = 711613, *h* = 1000, noise = 0, and aggregation = norm.

Calibration curves and the respective AUCEs for the considered models are shown in Figure 8, where a very good calibration is observed for systems with low noise and bias (*N* = 711613, *h* ≥ 100, noise ≤0.02, and mean aggregation). In fact, the artificial data set employed in this study is an ideal test case for calibration, because it features a controlled amount of noise and can be approximated with an arbitrary level of accuracy with a sufficient amount of data points and model degrees of freedom. We find that ensembling of d-MPNNs yields a well-calibrated measure of uncertainty for a prediction in this case. However, when adding larger errors from noise or bias, worse values for the AUCE are observed, since the total error of each prediction is now dominated by other contributions, thus impacting the correlation between ensemble uncertainty and true error. Model bias is often ignored as an error source but can significantly impact the ability of ensemble uncertainties to depict true errors. Data sets with a low amount of data points and large noise have been shown to lead to ill-calibrated models in the literature (for example the Lipophilicity data set in ref (47)), but the contribution of data coverage and other sources of bias is usually overlooked relative to the contribution of noise or variance. Even our artificial, noiseless and easy-to-learn data set leads to severely ill-calibrated models if the amount of training data is low (for example for *N* = 711 or 7116). The bottom panels of Figure 4 explain this failure: A low amount of training data leads to a bias-dominated model, where the total error is nearly exclusively due to bias, not variance. Because many chemical data sets are made of only hundreds to thousands of data points,^74,75^ we expect many deep-learning models to suffer from the calibration error caused by the data coverage model bias and should not be assessed using ensemble variance alone.

### Splitting and Data Leakage

So far, we have presented how noise, bias, and variance can lower the true or perceived performance of a model. An unsatisfactory model performance is detected easily, but the contributions of various error sources are often hardly distinguishable. We hope that the tools and insights presented above can aid scientists to better understand the sources of error in their models. This understanding can guide the next steps to optimize the model. Another possible pitfall comes when the performance of a model on the initial test set is much better than its performance on the actual molecules of interest, which can be hard to detect. In fact, models that reportedly perform well but fail in real-world applications are a major concern and setback within the machine learning community.^29,30^ In the following, we discuss two important reasons a model may seem to perform deceptively better than it actually does.

#### Generalization Performance

**Figure 9:**
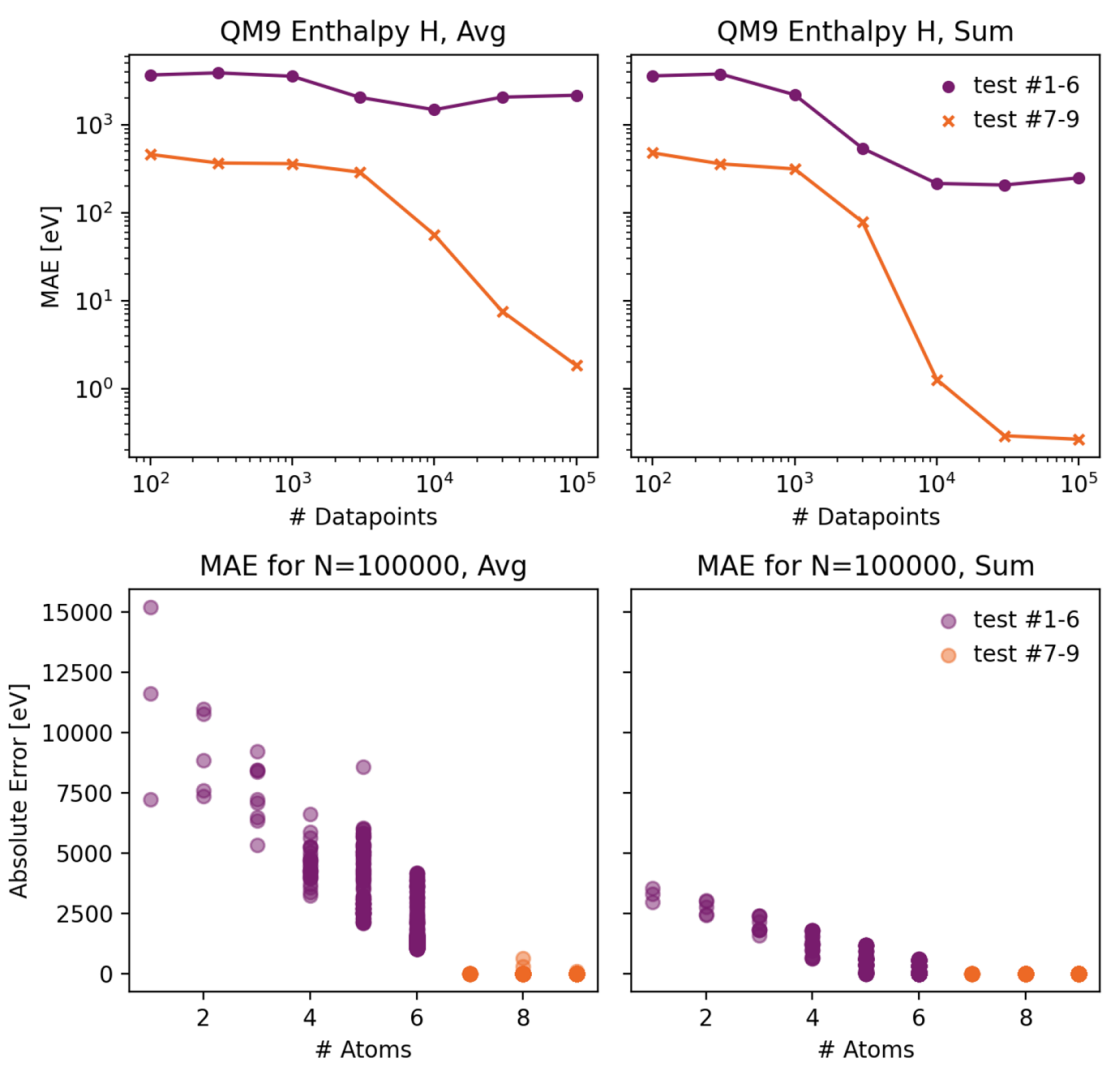
Top: Mean absolute errors as a function of the data set size for a test set containing only similar (7–9 atoms) or dissimilar (1–6 atoms) molecular sizes for a training set consisting of molecules containing 7–9 atoms, model using mean aggregation (left) or sum aggregation (right). Bottom: Dependence of the test set error of each data point on the molecule size for the model trained on 100,000 data points.

Limitations in the model architecture and representation can be easily overlooked if the data set only spans a small subset of chemical space. This may be the case for databases including only molecules with the same number of atoms or related chemical structures. As an example, we illustrate how a wrong choice in the aggregation function (which combines atomic into molecular embeddings) for the QM9 target enthalpy can be overlooked if the size of the molecules in the data set only spans a narrow range. To this aim, we split the QM9 data set into molecules with 1–6 atoms and 7–9 atoms. The machine learning models are then trained solely using the data set with 7–9 atoms. Figure 9 depicts the performance on test sets containing molecules of size 1–6 and 7–9 for a mean (top left) and sum (top right) aggregation function. For the test set containing similarly sized molecules, both aggregation functions lead to acceptable performances, and one might wrongly conclude that a mean aggregation function is a valid choice for an extensive target like the enthalpy. However, inspecting the test set performance on molecules of size 1–6 reveals that by using a mean aggregation function, the model does not gain any additional performance as more data is added. The bottom panels of Figure 9 depict the absolute errors for each data point in the test set as a function of molecular size. Here, the failure of the model utilizing mean aggregation becomes apparent: molecules with a different size produce absolute errors up to 4 orders of magnitude larger than molecules with a similar size because the model implicitly learns the average size of the molecules in the training set to circumvent the shortcoming of mean aggregation. We note that for sum aggregation, the extrapolation performance to differently sized molecules is by no means perfect (bottom right panel), but the model at least learns to generalize to some extent. In general, an extrapolation error is visible regardless of the model architecture. This extrapolation error can be assigned to bias by lack of data coverage for molecules with a lower molecular weight.

#### Test Set Contamination

**Figure 10:**
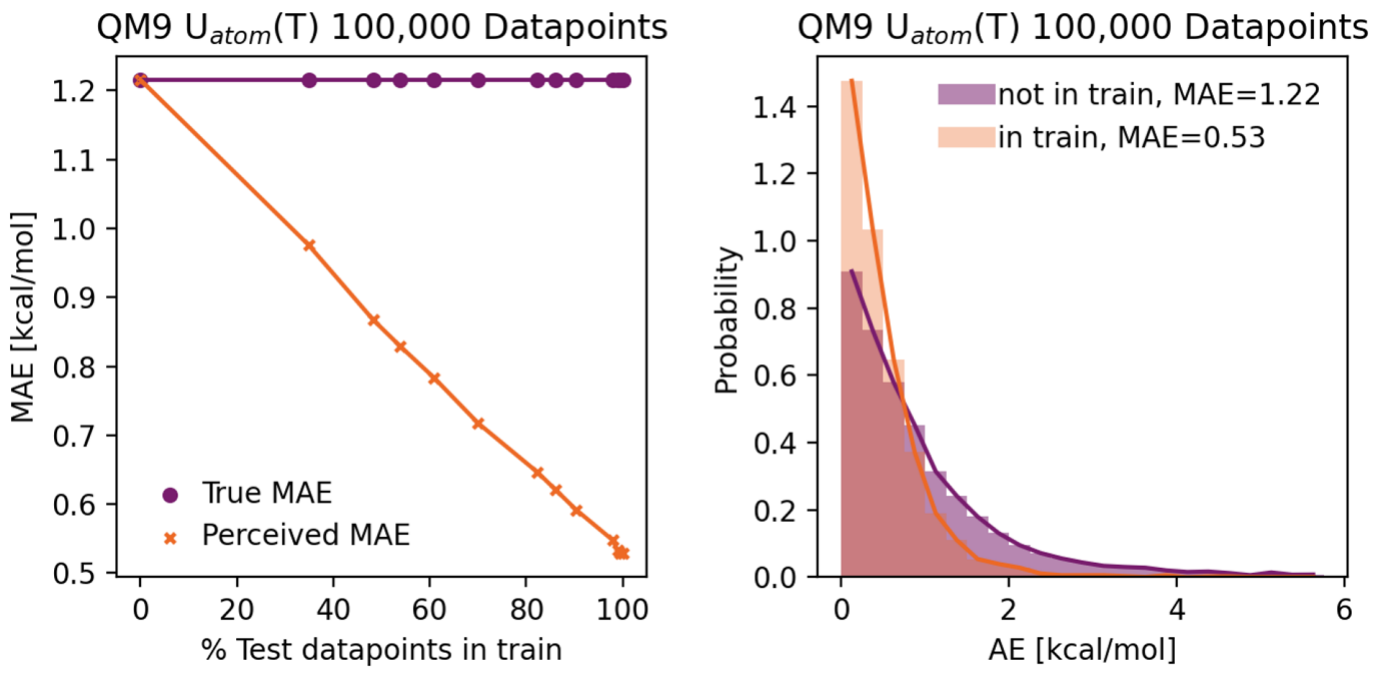
Left: The true versus the perceived test set error for 0–100% data leakage for d-MPNN models predicting *U*(T) at *T* = 0 K and *T* = 298 K on 100,000 data points, where data leakage corresponds to molecules in the test set that occur in the training set at a different temperature. Right: Distribution of absolute errors (AEs) for the test set, where the molecules do (“in train”) or do not (“not in train”) also appear in the training set.

Another prominent reason for a deceptively good performance is data leakage, where the test set is too similar to the training set. Rigorously splitting a data set into training, validation, and test sets is a crucial task that can be overlooked easily and may lead to drastically wrong reported performances.^76,77^ In the following, we showcase this pitfall by training a model of the QM9 target internal energy at temperatures *T* equal to 0 and 298 K. We treat the temperature as an input (in addition to the molecular graph) and train on the single property *U*(*T*). The temperature is appended to the aggregated molecular embedding (after the message-passing neural network, before the feed-forward neural network). If all data points are treated as independent, a massive amount of data leakage occurs since many molecules in the test set also occur in the training set, albeit at a different temperature. The left panel of Figure 10 depicts the true (only test set data points without leakage) and perceived (data points with and without leakage) performance for test sets with a different number of data points that are leaked. Depending on the constitution of the test set, different mean absolute errors are observed. For test sets with a large amount of leakage, the model appears to perform deceptively well. This perceived performance does not depict the intended application case, where the model is supposed to predict the internal energy of a new molecule outside the training set. The right panel of Figure 10 shows the distribution of the absolute errors of test data points that are held out during training versus data points leaked from the training set, where again the distribution of errors for leaked data points does not reflect reality, i.e. the distribution of errors for new, independent molecules.

The data leakage described above is easy to spot, but sometimes test set contamination occurs via more complex mechanisms. One example is our own ref (78) where a multitask model was trained on computational activation energies and reaction enthalpies of chemical reactions. Both forward and reverse reactions were included and treated as independent data points, so some of the reactions in the test set had their reverse counterpart in the training set. Out of several developed model architectures, one performed especially well, with accuracies close to chemical accuracy. However, this chosen architecture mainly excelled over the other potential architectures in exploiting the data leakage efficiently, leading to a seemingly good performance. When tested on independent reactions, however, the model produced errors about half an order of magnitude larger than reported. Only after removing the data leakage were we able to develop different architectures with better performance.^19^ In this case, test set contamination not only caused the reported test set error to be too low but also hindered model development and optimization. Similar cases were reported in the literature, where without a rigorous splitting strategy it was impossible to select the best architecture and parameters for reaction models.^76,77^ Splitting in systems involving multiple molecules that each may need rigorous splitting (*e.g., solute–solvent pairs*) creates an additional complication. In our previous work, we showed the importance of data splitting by excluding several solvents, solutes, and substructures from the training set.^70^ For chemical data sets, splitting according to molecular scaffolds^9,79^ or time-stamps^80^ can be an appropriate measure to prevent data leakage.^8,9,50,70,71,81−84^ In fact, the performance of many models was shown to drop significantly if evaluated on a more rigorous basis than simple random splits.^50,85−89^ However, scaffold splitting also comes with shortcomings, some of which have no easy remedy, as chemical space is inherently nonuniform and unbalanced.^90^ A detailed determination of optimal splitting strategies extends beyond the scope of this study. In general, we recommend rigorously splitting data sets when developing new models and paying increased attention to possible sources of data leakage.

## Discussion

Here, we discuss the main observed trends caused by noise, bias, and variance errors. Full diagnostic tools for quantifying the contributions of different error types do not yet exist. However, with the example of these trends, a dominant error type may be identifiable and treatable. We attempt to give practical advice on how to improve model performance in each of those cases.

Noise in a data set leads to a true loss in performance, as well as an additional and significant perceived loss in performance, which may cause a model seemingly to stop learning as soon as the true model error falls below the aleatoric limit. Whenever an asymptotic behavior of the model performance is observed in the learning curve, test noise should be considered as a possible cause. One example of this is shown in Figure 7 of ref (91). Further optimizing a model that has reached the apparent aleatoric limit is difficult, since a change in hyperparameters like the model size or architecture will lead to the same perceived test set error even though the true performance (measured by a clean test set) may have improved significantly. It is therefore important to construct test sets with a low amount of noise to develop and optimize high-precision models. We have recently shown the importance of a low noise test set for training neural networks to predict solvation free energies^91^ and aqueous solid solubilities,^11^ where cleaning the test set from large errors was necessary to develop a meaningful model. When there is reason to believe that a data set is affected by systematic noise, we recommend testing a model trained using mean-variance estimation or similar and comparing it against a simple model architecture.

For noiseless data sets, the reducible source of error is divided in a bias and variance term. Our recent application of Bayesian inference to ensembling allows users to quantify the error in both reducible contributions.^60^ By separating the contributions, it becomes possible to prioritize efforts between reducing model bias and model variance. Reducing the model bias error is tedious and requires user experience. Bias can be reduced by adding more data and by choosing the best possible molecular representation, model architecture, and set of features to relate the molecular structure to the target property. These challenges are particularly common in chemistry; the vast chemical space makes data size and coverage a large source of error compared to other fields of research, where many chemical structures are unique or under-represented in (experimental) data sets. The representation of molecules inside machine learning is without question one of the main challenges in chemical property prediction today. In other fields of machine learning such as computer vision or natural language processing, the size of an image or a sentence does usually not scale with the output target. For example, the number of words in a sentence or letters in a word do not tell us about its meaning, conveyed information, or sentience. In contrast, for extensive properties, the size of a molecule changes its properties significantly, so that representations and architectures developed in other fields of research must be properly adapted to chemical applications. Careful consideration of several representations and selecting the most appropriate for the target property is crucial in reducing the bias error. Properties may not always be easily delineated between intensive and extensive, so we recommend choosing extensive aggregations in chemical systems when in doubt.

Finding optimal features is important for medium-sized data sets (bias error by featurization reduces when the relation between structure and property can be learned from more data). The customization of atomic and molecular features for a task at hand is an important aspect of model optimization even for deep learning models because the optimal features are not selected automatically. Despite these insights being rather expected, we find that often not enough attention is paid to featurization when building new models.

The variance error can be reduced by, for example, ensembling, regardless of the other sources of error in the model. There is a trade-off between the gain in model performance and the computational load of training more models. For a quick assessment, we recommend training an ensemble of five models and using the slope of the performance improvement from subsets of the five models to estimate whether additional models should be added. For a more extensive estimation of possible gains from ensembling, we recommend our method for projecting the expected error of different ensemble sizes from ref (60). Depending not only on the task, data set, and architecture but also the availability of computation power and intended use of the model, a different number of ensemble models will lead to the best trade-off between performance and computational workload.

Different error types may be caused by a single source. In our treatment of the bias and variance error present in the noiseless data set, we note that bias and variance errors both increase in circumstances with small data set sizes and small model sizes. A single source, whether it is data sparsity or a problem of model architecture choice, can manifest simultaneously as both kinds of errors. Similarly, our experiments with the addition of controlled noise to a data set showed separate reducible and irreducible errors depending on whether the noise was in the test set or used in training, an example of noise addition leading to both noise and bias errors. Interactions, trends, and correlations between error types will exist in real data sets that go beyond simple error type assignment.

In addition to the specific error types addressed, we highlight the importance of avoiding data leakage, which unfortunately is rather common in chemical data sets. Leaked data and the associated overly optimistic reported model performance hamper the development of new models severely, reduce the confidence in machine learning models, and delay their application to real world scenarios. We therefore urge the reader to pay increased attention to data splitting when developing models on new data sets.

In many cases, the uncertainty quantification tools that we have discussed here are used in concert with uncertainty calibration techniques. Various calibration methods exist for adjusting the magnitude of uncertainty predictions in the context of regression models.^92−94^ Application of these methods often works by scaling the uncertainty predictions made by a model to match the real errors observed in a held out calibration data set. Application of calibration methods may serve to improve some uncertainty evaluation metrics, such as miscalibration area, while still providing uncertainty quantifications with functional shortcomings. Two useful evaluation metrics to consider for the suitability of uncertainty quantification calibrations are sharpness and dispersion, as discussed in the context of materials data sets by ref (95). Sharpness refers to the average level of precision predicted by a model, in that a model that is accurately represented as low uncertainty is better than a model that is accurately represented as high uncertainty. Dispersion refers to the ability of a model to distinguish between high and low uncertainty predictions within a data set. Failures to account for error types using the appropriate tools may be compensated for with calibration techniques but doing so inappropriately will often lead to poor sharpness and dispersion. In the systematic noise section of this paper, the failure of ensemble variance to distinguish between error regimes is an example of poor dispersion, even when scaled to a calibrated level. We caution the reader to apply calibration methods carefully and check their validity using multiple evaluation metrics.

## Conclusion

We have demonstrated the role of noise, bias, and variance for the perceived and true performance of machine learning models, focusing on chemical applications. Understanding the possible sources of errors in an underperforming model is an important prerequisite to identifying potential improvements.

Noise inherent to data is commonly found as experimental uncertainty in chemical data sets. The presence of noise has a different effect on the perceived model performance depending on whether it is found in the training and/or test set. Noise in the test set leads to an observed aleatoric limit and can cause an underestimation of the true model performance. We furthermore highlighted challenges in predicting properties of molecules, such as the choice of size-conserving representations and architectures for the prediction of size-extensive targets. Limitations in the data set size, model architecture, or representations can cause the overall model error to be dominated by the contributions of model bias. We discuss ensembling as a reliable method to reduce model variance error and the value of using statistical tools to evaluate the portion of the error due to variance. However, in situations where noise or bias error dominate, ensembling cannot be used to correct for those errors, and ensemble variance becomes ineffective at estimating whole model uncertainty. Lastly, we showcased the effects of splitting and data leakage when assessing the real-world performance of a model and strongly advise researchers to pay close attention to meaningful data splits avoiding leakage.

In summary, machine learning is a valuable and important tool to predict physicochemical properties but can suffer from error sources uncommon to other fields of research. Increased attention should be paid to noise and bias from data coverage, model architecture, and representation to identify and remedy shortcomings of chemistry-related deep learning models concerning their performance and uncertainty calibration.
